# Environmental and economic determinants of temporal dynamics of the ruminant movement network of Senegal

**DOI:** 10.1038/s41598-023-40715-3

**Published:** 2023-09-02

**Authors:** Katherin Michelle García García, Andrea Apolloni, Alessandra Giacomini, Mamadou Ciss, Mathioro Fall, Adji Marème Gaye, Elena Arsevska, Asma Mesdour, Etienne Chevanne, Fabrizio Rosso, Eric Cardinale, Cécile Squarzoni Diaw, Ismaila Seck, Mbargou Lo, Alexis Delabouglise

**Affiliations:** 1 CIRAD, UMR ASTRE, Montpellier, France; 2 CIRAD, UMR ASTRE, Univ Montpellier, INRAE, Montpellier, France; 3 Department of Biosciences, Swansea University, Swansea, SA2 8PP UK; 4 Institut Sénégalais de Recherches Agricoles/Laboratoire National de l’Elevage et de Recherches Vétérinaires, BP 2057, Dakar-Hann, Sénégal; 5 Direction des Services Vétérinaires, Dakar, Sénégal; 6 Food and Agriculture Organization of the United Nations, The European Commission for the Control of Foot-and-Mouth Disease, Rome, Italy; 7 Food and Agriculture Organization of the United Nations, One Health and Intelligence and Early Warning Office, Rome, Italy

**Keywords:** Risk factors, Statistics, Ecological epidemiology

## Abstract

Our understanding of the drivers of the temporal dynamics of livestock mobility networks is currently limited, despite their significant implications for the surveillance and control of infectious diseases. We analyzed the effect of time-varying environmental and economic variables—biomass production, rainfall, livestock market prices, and religious calendar on long-distance movements of cattle and small ruminant herds in Senegal in the years 2014 and 2019. We used principal component analysis to explore the variation of the hypothesized explanatory variables in space and time and a generalized additive modelling approach to assess the effect of those variables on the likelihood of herd movement between pairs of administrative units. Contrary to environmental variables, the patterns of variation of market prices show significant differences across locations. The explanatory variables at origin had the highest contribution to the model deviance reduction. Biomass production and rainfall were found to affect the likelihood of herd movement for both species on at least 1 year. Market price at origin had a strong and consistent effect on the departure of small ruminant herds. Our study shows the potential benefits of regular monitoring of market prices for future efforts at forecasting livestock movements and associated sanitary risks.

## Introduction

Network analysis of livestock movements has been recently established as one of the most valuable analytical tools for guiding the surveillance and control policies of livestock transboundary animal diseases (TADs). In parallel to the accumulation of evidence of the predominant role played by the movement of livestock in the diffusion of pathogens over long distances and in the extension of livestock epidemics^1–3^, there is a growing recognition of the usefulness of collecting, centralizing and analyzing mobility data for targeting surveillance and intervention policies^4,5^. In most cases, however, mobility data are collected only sporadically or during specific periods of the year and provide a partial and static representation of the networks that fail to account for their intrinsic dynamic nature. This is a significant caveat since the evolution of network structures over time has significant implications for epidemiological processes^6^. Animal movements, either for commercial reasons or production needs, are affected by a variety of factors, from the fluctuation of demand and seasonal variation of consumption of livestock products to changes in the environmental conditions of farming^7,8^. The need to explore the drivers of temporal dynamics of livestock networks is especially pressing in low- and low-middle-income countries where live animal mobility is vital to a large fraction of the livestock industry and where reliable identification systems of domestic animals are missing and livestock trade is mostly informal^9,10^.

The arid region of Sahel, extending from Senegal and Mauritania on the West, to Chad and Sudan on the East, is a major production area for ruminants, essentially cattle, sheep and goat. The large majority of ruminants are kept by pastoralist and agro-pastoralist households practicing extensive farming with little investments in animal housing and feed^11^. Animals in these systems contribute to households’ livelihood in diverse ways, as they provide stable income, subsistence food and social capital^12^. Ruminant farmers in Sahel not only supply their domestic markets but also the densely populated countries bordering the Gulf of Guinea^13,14^. However, livestock productivity is constrained by some factors, infectious diseases being among the major ones^15^. Major TADs of ruminants include Foot and Mouth Disease^16^ and Peste des Petits Ruminants^17^ while emerging and zoonotic TADs, such as Rift Valley Fever, threaten the public health and economic and political stability of the Sahelian countries^18,19^.

Long distance livestock movements in Sahel can be classified into two broad categories: trade movements and pastoral mobility, the latter also referred to as transhumance mobility. Trade movements of live animals play a fundamental role in the supply of livestock products to the major consumption centers, in the absence of appropriate means of storage and transportation of meat and the sale of livestock represents up to 97% of pastoral households’ income^14^. Trade and transport of animals are primarily mediated by traders who buy and sell animals at dedicated marketplaces^20^. The frequency of commercial movements of ruminants is driven by the demand for animal products and by environmental conditions, as farmers tend to sell their animals to reduce their herd size during times of shortage of pasture, in the dry season^21^. Ruminants also play a significant cultural role with rams and cattle being ritually slaughtered in Senegal during the Tabaski and Magal de Touba celebrations respectively^22^. According to Turner and Schlecht^23^, pastoral mobility is primarily aimed at optimizing livestock nutrition. Indeed pastoral farmers move their herds over a long distance when they anticipate the benefit of finding better grazing areas to exceed the travel cost, and they use well-known paths and resting points to minimize the risks associated with the journey. According to Adriansen^24^, reasons for Senegalese pastoral farmers to move their herds include southward migration towards areas where the rainfall is expected to start first, at the very beginning of the rainy season, search for better pastures, usually during the dry season, and adverse events at the place of departures, such as bush fire, disease outbreak or shortage of water. In areas combining extensive livestock farming and cultivation, an additional reason for moving livestock is the avoidance of conflicts with crop producers, particularly in the rainy season^23^. Therefore, pastoral mobility can be viewed as a form of risk management adapted to the specific conditions of Sahel, where farmers are confronted with risks of droughts, disease outbreaks, criminality, and competition with crop farms, all these factors increasing under the combined effect of climate change and political instability^25^.

Static networks of ruminant’s movements were analyzed in several West African countries, including Togo^26^, Mauritania^27^ and Burkina Faso^28^, and pointed to the high frequency of transboundary movements and associated disease transmission risk. The data used in these studies are composed of official movement registries based on the delivery of sanitary permits by Veterinary Services to the owners of animals to authorize the travel, or obtained from surveys conducted with a sample of livestock traders or farmers, usually at markets. In both cases, the used datasets capture only a limited fraction of the actual movements, but they can be used to identify reliable predictors of network edges—i.e., the existence of at least one animal movement over a time period between pairs of defined locations—through statistical modelling^5^. Predictive models fitted to static livestock network data provided valuable insights of the geographical and economic factors shaping livestock networks. Nicolas et al.^29^ applied a gravity model to the cattle, small ruminant and camel movement network data based on the official movement registry of Mauritania. They demonstrated that livestock movements tend to be directed from areas with high sheep density and low human population towards area with low sheep density and high human population. Belkhiria et al.^30^ analyzed herd movement data obtained from a survey of itinerant farmers conducted in the Ferlo region, in northeast Senegal, using an exponential random graph model. The analysis showed that the lack of water and the presence of a livestock disease are likely drivers of outgoing livestock movements, and that farmers preferentially travel to locations with livestock markets.

To the best of the authors’ knowledge, no attempt was made to analyze the contribution of time-varying factors, like climatic or market conditions, to the temporal dynamics of livestock networks. Fulfilling this gap would have significant implications, since the identified effects could be used for forecasting, to some extent, the evolution of networks over time and for better allocating disease control interventions, like sanitary controls and vaccination on livestock herds, and surveillance in space and time^31^. In the present study we analyzed the ruminant mobility data of Senegal provided by the National Veterinary Services (DSV) to understand how, and in which limitations, socio-economic and environmental factors shape the temporal pattern of mobility in the country. The choice of Senegal is justified by the economic importance of livestock production and mobility in this country^32^, its position at the crossroad of international transhumance and trade movements pathways, and the gradient of climatic conditions existing between its northern semi-arid and its southern tropical part^33^. Additionally, a systematic recording of animal movements and market prices have been implemented for some years by the DSV, and by the Vulnerability Analysis and Mapping (VAM) office of the World Food Program (WFP) respectively. These recordings were the source of the data used in our analysis. The present study aimed at (1) describing the spatio-temporal dynamics of variables that are potential drivers of ruminant movements and (2) modelling the effect of these covariates and of the calendar on the movements of cattle and small ruminant herds in Senegal.

## Material and method

### Data acquisition

The livestock mobility network was reconstructed from data provided by the DSV for years 2014 and 2019^34^: local veterinary officers deliver sanitary permits, called “Laissez passer Sanitaires” (LPS), to livestock owners, which is a legal requirement for moving livestock herds between communes. The LPS contains date, origin and destination (commune, department, region, country) of movement, the number of animals, their species, and the means of transportation, but does not include information on individual animals like breed, sex or age. In 2014 and 2019, the copies of the LPS were gathered and their information was centralized in a dataset by the central office of the Veterinary Services. These datasets were used to compute the number of livestock movements recorded between specific departments of Senegal during each month of those two years. In 2019, the dataset was completed by information collected through ad-hoc survey at border points (called “PIF”) to include information about international movements.

Several time-varying environmental and anthropogenic covariates were used to inform the models. Rainfall data was obtained from the Global Precipitation Climatology Center (GPCC) in NetCDF format with a 0.25° grid resolution and in mm/mm^2^ unit^35^. The rasterized rainfall data was converted into monthly average cumulative rainfall per department. The biomass data was downloaded from Copernicus Global Land Service using the dry matter productivity indicator^36^. Dry matter productivity was used as an indicator of biomass production. It represents the dry biomass increase of the vegetation in a given area, in kilograms per hectare per day. The data was provided in a rasterized form, with a resolution of 333 m, which was converted into average monthly dry matter productivity per department.

Live cattle and small ruminant market prices were provided by the VAM office of WFP^37^. The VAM office monitored the price of 53 markets distributed across the national territory, in West African CFA (“communauté financière africaine”) currency. At specific dates, the live animal prices applied in a varying number of commercial transactions were collected and the average price was considered as the reference price of the day. This price aggregation did not account for the characteristics of the sold live animals (e.g. age, weight, sex, breed). Monthly average prices were computed in case more than one price collection occurred in markets per month.

### Analysis of the explanatory variables’ dynamics

A principal component analysis (PCA) was used to describe the patterns of variation of the explanatory variables (rainfall, biomass production, market price) over time and the degree of heterogeneity of these dynamics across locations. For biomass and rainfall, the considered locations were departments. Aggregated values of biomass production and rainfall were available at the department level on every month in all 45 departments of Senegal in 2014 and 2019. For market prices, 14 out of 45 departments had no market where livestock price was recorded. In 2014, no price data was available for January in all markets, and a price data was available for February in only 5 markets. Given the limited coverage of the market price monitoring at department level and the high number of missing observations—i.e., months where no price data was recorded in the monitored markets—we did not apply the descriptive analysis at the department level, but rather at the level of markets were price data was recorded with no interruption from March to December. The PCA was therefore applied on the price time series of 12 markets and 15 markets in 2014 and 2019, respectively. Locations were considered as variables and months as observation points. The variables were centered and standardized in order to focus the analysis on the variables’ trend over time rather than their average and variance. The value of the first dimension returned by the PCA at each month was considered as the dominant dynamic pattern. If more than 70% of the variance was explained by the first dimension the dynamics were considered as homogeneous across locations. Otherwise, an agglomerative hierarchical clustering approach was applied to divide the locations into clusters. The clustering process used the Ward’s variance minimization method^38^ applied to a dissimilarity matrix between locations computed using the Gower distance coefficient^39^. The difference between the average distance separating pairs of locations belonging to different clusters and pairs of locations belonging to the same cluster was then computed. To evaluate the statistical significance of this difference, a permutation test was used: the same difference was evaluated after randomly allocating locations to clusters in 10,000 iterations. The p-value corresponded to the proportion of iterations where the simulated absolute difference was equal or higher than the observed absolute difference.

### Predictive models of the dynamics of ruminant herd movement

A statistical model was built to test the effect of four variables on the likelihood of cattle and small ruminants’ movements: biomass production, rainfall, market price of the corresponding species and calendar month. The chosen unit of observation was a pair of origin and destination department (later referred to as “inter-department link”) on a given month and the observed dependent variable was the count of herd movements from department or origin to department of destination on the considered month. There are 45 departments in Senegal, so 1980 inter-department links. The aggregation at the level of other administrative units, namely region (n = 14) and district (“arrondissement”) (n = 133), would have resulted in a low number of observations and loss of information or an excessive dispersion of movement counts respectively.

Biomass production, rainfall, and market price can influence the decisions to move herds in different ways. Owners of ruminants may base their decision on the value of these variable at the department where they are currently located (the department of origin) as well as at the department of intended destination. Furthermore, they may take into consideration the observed absolute value of the hypothesized variable or rather its change from one month to the next. Independently of the tested covariates, some inter-department links have a higher count of herd movements because of their intrinsic properties, for example the ones connecting production areas (with a high domestic ruminants density) and consumption areas (with a high human population density)^29^. This justifies the inclusion of inter-department links as individual fixed effects in the predictive model. In addition, the relationship between the hypothesized variables and the calendar months and the likelihood of herd movements is likely to significantly depart from linearity. To address these constraints a generalized additive modelling approach (GAM) was chosen^40^. The structure of the model is described by the following equation:

$$log\left( {\mu_{ijt} } \right) = \mathop \sum \limits_{k} f_{O}^{k} \left( {X_{it}^{k} } \right) + \mathop \sum \limits_{k} f_{D}^{k} \left( {X_{jt}^{k} } \right) + g\left( t \right) + \gamma_{ij} + \in_{ijt}$$

With $\mu_{ijt}$ the number of herd movements recorded between department $i$ and department $j$ at time $t$; $f_{O}^{k}$ and $f_{D}^{k}$ fitted thin-plate spline functions of the predictive variable $k$ at the department origin (*O*) and destination (*D*) respectively^41^; $X_{it}^{k}$ and $X_{jt}^{k}$ the values taken by the variable $k$ at month $t$ in department $i$ and department $j$ respectively; $g$ the fitted spline function of the calendar month; $\gamma_{ij}$ the fixed effect of the inter-department link $i$ to $j$; and $\in_{ijt}$ the residual error term. The predictive variables are: department average rainfall on the previous month (t−1); difference between department average rainfall on current (t) and previous month (t−1); department average biomass production on the previous month (t−1); difference between department average biomass production on current (t) and previous month (t−1); regional average market price on the previous month (t−1); difference between regional average market price on current (t) and previous month (t−1).

Because of the large number of departments with no market price monitoring, we used the aggregated market price at the regional level rather than departmental level as a covariate in the GAM model. Yet, missing price observations remained in the dataset. In 2019, there was at least one month with missing price data in 4 out of 14 regions. In 2014, no price data was available for January in all regions, and a price data was available for February in only one region. Also in 2014 no price data was available for Dakar and Sedhiou regions in every month and there were missing data from March to December in 5 out of the 12 other regions.

Given the large number of “zero values”—i.e. observations with no recorded movements—the distribution was modelled as a hurdle Poisson. With this modelling approach the complimentary log–log of the probability of absence of observations is modelled as a linear function of $log\left( {\mu_{ijt} } \right)$ while the probabilities of counts of herds higher than zero are modelled by a truncated Poisson distribution with parameter $\mu_{ijt}$. Given the model structure, the observation points with missing values of regional market price in the current or previous months at the department of origin or department of destination were removed. The distribution of rainfall being highly skewed, the monthly rainfall covariates were square-root transformed before inclusion in the model. The dimension of each spline must be specified in the model. For the splines of the tested variables, the dimension was fitted through a dimension penalization process while for the calendar month the dimension was fixed. A range of dimension values for the calendar month spline (from 0 to 10) were tested and the right dimension was considered to be the one minimizing the Akaike Information Criterion (AIC). Arguably, the number of livestock movements in one given month may influence the number of livestock movements in the following month, independently of the tested variable, because farmers or traders may imitate each other. Therefore, we tested the presence of time autocorrelation at the level of inter-department links by fitting two linear regression models on the deviance residuals, with a fixed constant effect and with and without an AR-1 time autocorrelation term inside each inter-department link. We tested the difference between the two model fits with a log-likelihood ratio test. Residual autocorrelation was confirmed if the inclusion of the time autocorrelation term significantly improved the model fit. Excessive multi-collinearity between covariates was assessed by estimating their variance inflated factor (VIF).

### Computing material

Data processing, statistical analysis and graphical representations were performed with R version 3^42^. The following R-packages were used for analytical purpose: “usdm”^43^ for exploring multi-collinearity between covariates by computing the variance inflated factor; “mgcv” for fitting General Additive Models^44^, the basic functions of R for performing PCA, and “cluster” for clustering livestock locations on the basis of PCA results^45^. The rasterized rainfall and dry matter productivity data were converted into monthly cumulative rainfall and average dry matter productivity per department using QGIS software^46^.

## Results

The evolution of rainfall and biomass aggregated at departmental level is displayed in Figs. S1 and S2 respectively. The availability and evolution of the market price aggregated at regional level is displayed in Fig. S3 for cattle and Fig. S4 for small ruminants.

### Patterns of environment and market price dynamics

**Figure 1:**
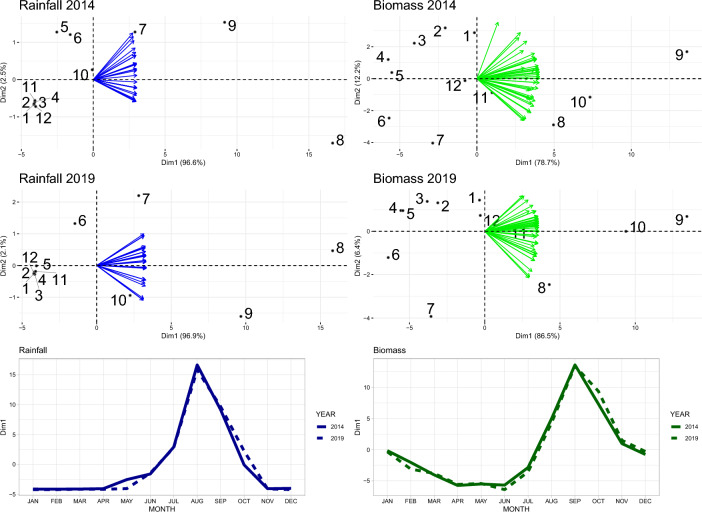
Results of the principal component analysis performed on the rainfall (left) and biomass production (right) aggregated at month and department level in 2014 and 2019. The top and middle graphs biplots represent the values taken by the departments (arrows) and months (dots numbered from 1 to 12 in chronological order) on the first and second dimensions (the X and Y axis respectively). The bottom graphs represent the value taken by the successive months on the first dimension (which explains most of the data variance) in 2014 (plain line) and 2019 (dashed line).

The results of the PCA performed on environmental variables are displayed in Fig. 1. The patterns of variation of rainfall and biomass were highly homogeneous across departments, with a first dimension explaining more than 75% of the data variance and indicating (1) a rainfall level alternating between a dry season, with very low, if not absent rain from November to April and a rainy season starting in May–June and peaking in August; (2) a biomass availability alternating between a minimal level in the March-June period and a maximum level in September.

**Figure 2:**
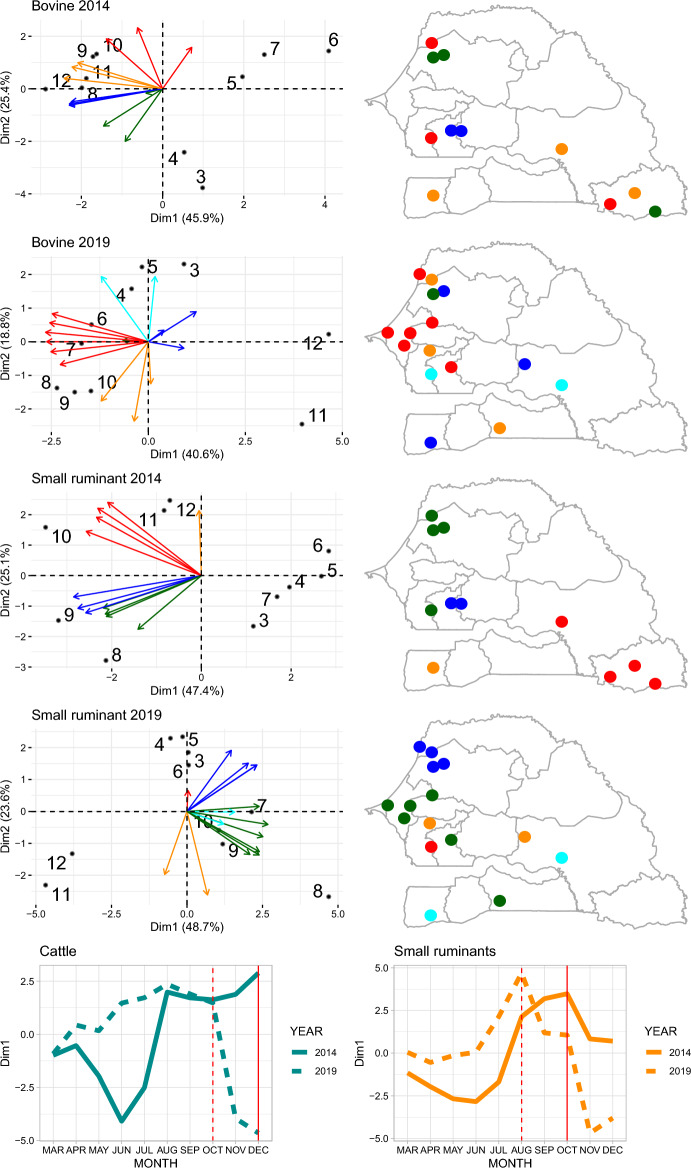
Results of the principal component analysis performed on the market price of cattle and small ruminants in 2014 and 2019 in a selection of monitored markets. The left hand biplots represent the values taken by the markets (arrows) and months (dots numbered from March to December in chronological order) on the first and second dimensions (the X and Y axis respectively). The color of the arrows corresponds to the cluster the markets were attributed to. The right hand maps represent the geographical location of the markets, with colors corresponding to their attributed cluster. The bottom graphs represent the value taken by the successive months on the first dimension in 2014 (plain line) and 2019 (dashed line) for cattle (left) and small ruminants (right). The plain and dashed vertical red lines correspond to the months of important religious celebrations where cattle and small ruminants are traditionally consumed in 2014 (plain) and 2019 (dashed) (Magal de Touba for cattle, Tabaski for small ruminants). Administrative boundaries were drawn using the GADM database of Global Administrative Areas (www.gadm.org).

Contrary to what was observed for environmental variables, patterns of price dynamics substantially differed across monitored markets (Fig. 2). In all four cases—cattle and small ruminants in 2014 and 2019—the two first PCA dimensions explained more than 59% of the data variance and the first dimension more than 40% of the data variance. As illustrated in Fig. 2, the predominant pattern of price dynamics (i.e. the first dimension) corresponds to a price fall in June 2014 and in November–December 2019 for both species, a price peak for small ruminants at the time of Tabaski for both 2014 and 2019 and relatively high cattle prices in the five months preceding the Magal de Touba celebration. In 2014, the live cattle price reached its peak at the time of Magal de Touba (December) in 5 out of 12 markets and the live small ruminant price reached his peak at the time of Tabaski (October) in 6 out of 12 markets.

Markets in 2014 and 2019 were classified into 4 and 5 clusters respectively, through agglomerative clustering following the Ward’s variance minimization method, applied to the values taken by markets on the two first dimensions. The geographical location of markets belonging to different clusters is displayed in Fig. 2. The average geographical distance between markets belonging to the same cluster was smaller than the one of markets belonging to different clusters. However, this difference was significant only for small ruminants (a permutation test p value < 0.01 in both years) and not for cattle.

### Description of the mobility data

The number of observations kept in the predictive models was 265 and 777 for cattle in 2014 and 2019 respectively, and 387 and 1051 for small ruminants in 2014 and 2019 respectively. The mean number of recorded herd movement per observation was 1.5 and 3 for cattle in 2014 and 2019 respectively and 1 and 1.4 for small ruminants in 2014 and 2019 respectively. The number of recorded movements was substantially dispersed. The proportion of observations with absence of movement was 74% for cattle both in 2014 and 2019 and 75% and 77% for small ruminants in 2014 and 2019 respectively. The maximum number of recorded movements in one observation was 79 and 151 for cattle in 2014 and 2019 respectively and 33 and 109 for small ruminants in 2014 and 2019 respectively.

**Figure 3:**
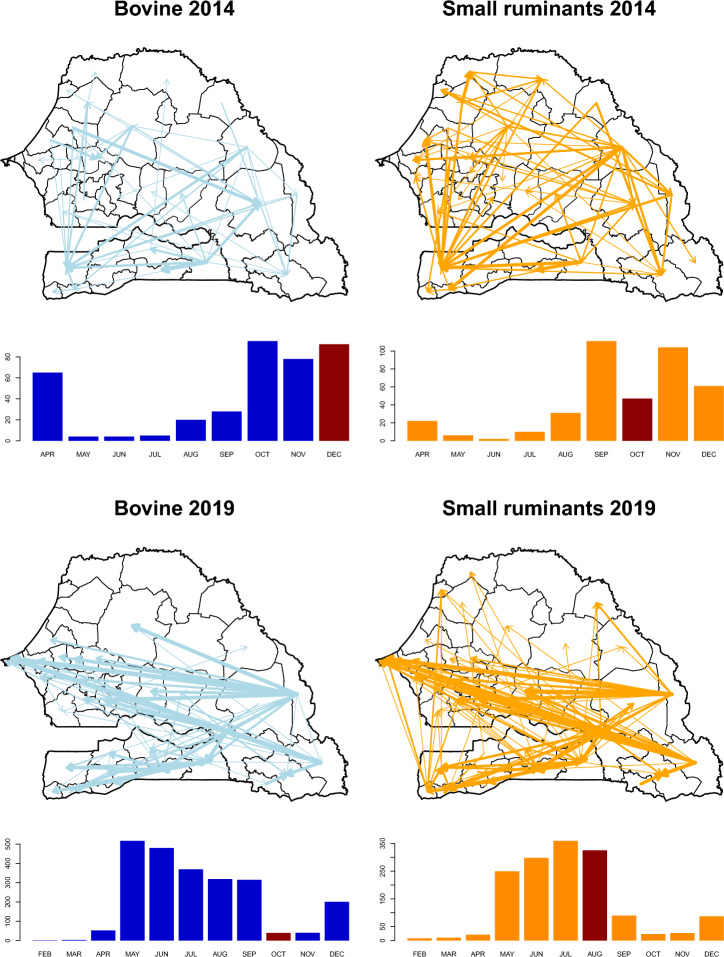
Graphical representation of the spatial and temporal distribution of the number of the recorded cattle herd (left) and small ruminant herds (right) movements between departments of Senegal in 2014 (top) and 2019 (bottom). The inter-departments links on which at least one movement was recorded are displayed on the maps with arrows’ width proportional to the logarithm of the number of recorded movements in the year. The evolution of the number of recorded movements through time is displayed in the bar plot, with the number of recorded movements throughout the country and the calendar month in Y and X axis respectively. Important religious celebrations (Magal de Touba for cattle, Tabaski for small ruminants) are indicated with a brown bar. Administrative boundaries were drawn using the GADM database of Global Administrative Areas (www.gadm.org).

The variation of the number of recorded herd movements included in the analysis in space and in time is displayed in Fig. 3. It can be visualized in more details in Figs. S5 and S6. The distribution of recorded herd movements is geographically balanced across Senegal in 2014, while in 2019 the large majority of recorded herd movements originated in the southeastern part of the country, with 99% of cattle movements and 95% of the small ruminant herd movements originating in Kedougou, Kolda and Tambacounda regions, all located in the Southeast. The number of recorded movements varied substantially over time, and peaks did not necessarily correlate with the celebration of religious festivals where ruminants are traditionally consumed—Tabaski for sheep, Magal de Touba for cattle.

### Predictive model results

The fitted GAM model characteristics are displayed in Table 1. The variables “rainfall at destination in the previous month” and “change in rainfall at destination” were removed from the model due to their excessive correlation with the “rainfall at origin in the previous month” and “change in rainfall at origin” respectively. The models predict less than 0.5 cattle movements for 86% of observations with no recorded cattle movements in 2014 and 2019 and they predict less than 0.5 small ruminant movements for 82% and 87% observations with no recorded small ruminant movements in 2014 and 2019 respectively. No cattle movements were recorded in 85% and 86% of observations where the number of predicted cattle movements is less than 0.5 in 2014 and 2019 respectively, and no small ruminant movements were recorded in 89% and 90% of observations where the number of predicted small ruminant movements is less than 0.5 in 2014 and 2019 respectively. For observations with no registered movements, the models predict a maximum of 7.3 and 57.2 cattle movements in 2014 and 2019 respectively and 7.6 and 13.3 small ruminant movements in 2014 and 2019 respectively. The concordance between predicted and observed value is illustrated in Fig. S7. No significant time autocorrelation was found in the model residuals.

**Table 1:**
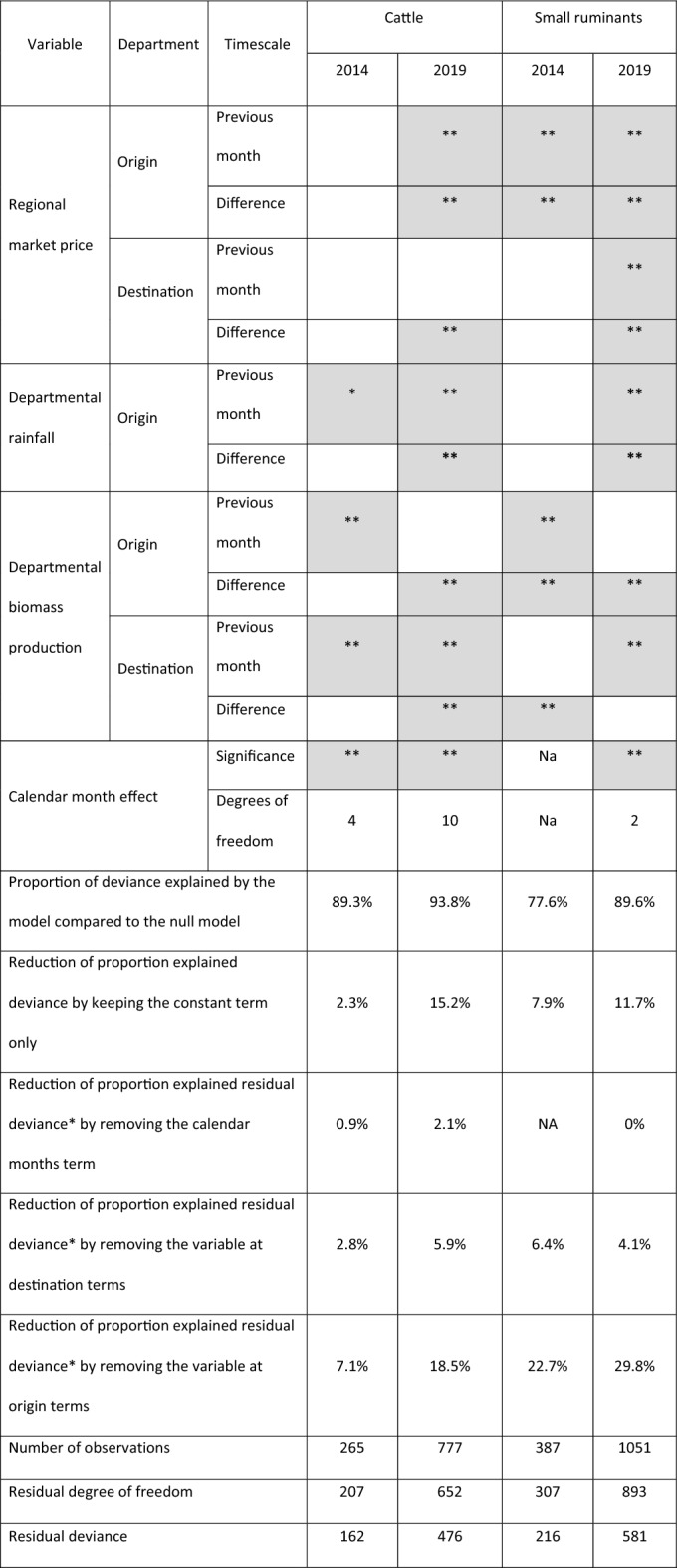
Characteristics of the fitted GAM hurdle Poisson models for predicting the monthly count of recorded inter-department cattle and small ruminant herd movements in 2014 and 2019.

Significant terms are indicated in gray color (*p value < 0.05, **p value < 0.01).

*Residual deviance here refers to the deviance not explained by the model with constant terms only (the interdepartmental links).

The proportions of deviance explained by the models are 89% and 94% for cattle in 2014 and 2019 respectively and 79% and 89% for small ruminants in 2014 and 2019 respectively (Table 1). The contribution of the tested time-varying variables to the model fit (the reduction of the percentage deviance explained when removing these variables) was limited (maximum 15.2%), and particularly low for cattle in 2014 (only 2.3%). The tested time-varying variables at the department of origin (biomass, rainfall and market price) were more influential than the time-varying variables at destination and the calendar month effect, especially in small ruminants, their removal reducing the explained residual deviance by nearly 23% and 30% in 2014 and 2019 respectively.

**Figure 4:**
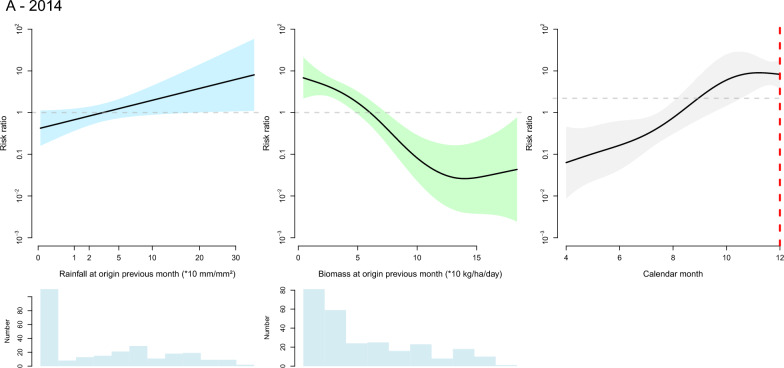

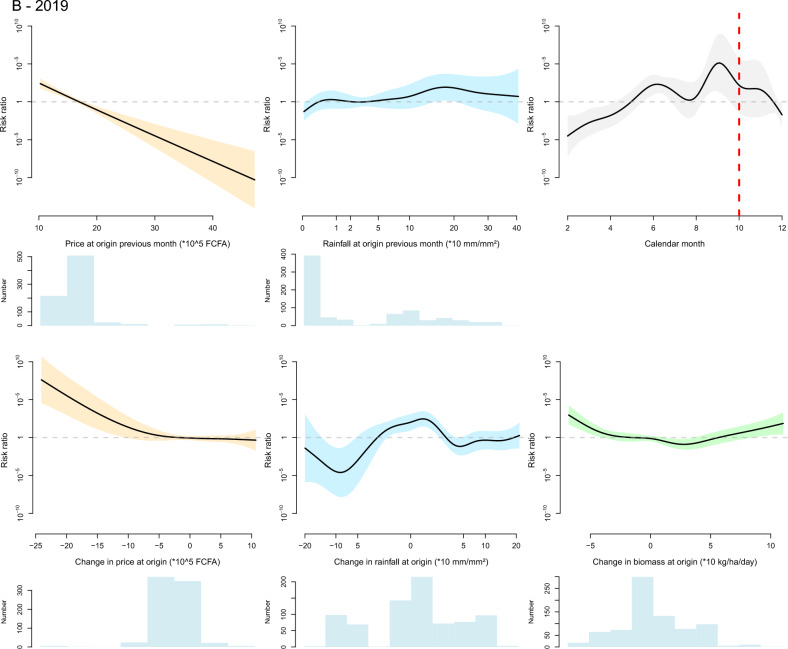
Graphical representation of the fitted thin plate spline functions of the effect of the selected variables on the likelihood of bovine herd movement along a defined inter-department link on a defined month in 2014 (**A**) and 2019 (**B**). Black lines are spline functions linking the tested variable to the the risk ratio of herd movement, and blue bands correspond to the 95% confidence interval. Blue histograms at the bottom of each graph represent the distribution of the selected variable (the y axis is the count of observations). On the graph displaying the effect of calendar month, the dashed red vertical line corresponds to the Magal de Touba celebration.

**Figure 5:**
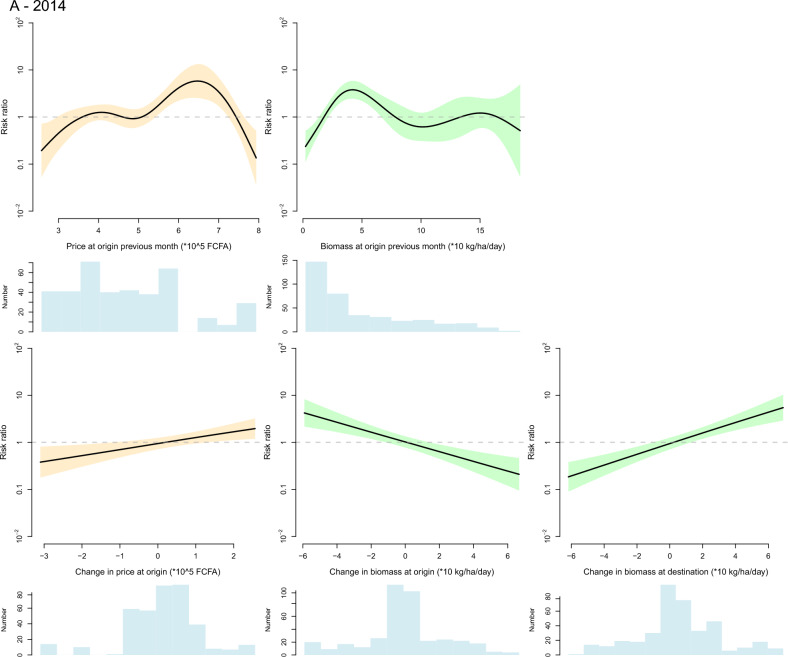

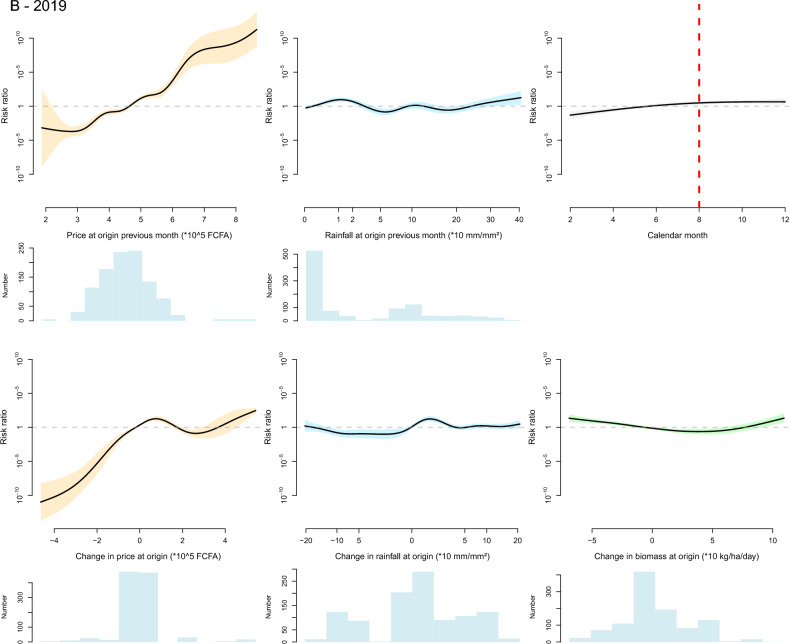
Graphical representation of the fitted thin plate spline functions of the effect of the selected variables on the likelihood of small ruminant herd movement along a defined inter-department link on a defined month in 2014 (**A**) and 2019 (**B**). Black lines are spline functions linking the variable to the risk ratio of herd movement, and colored bands correspond to the associated 95% confidence interval. Blue histograms at the bottom of each graph represent the distribution of the selected variable (the y axis is the count of observations). On the graph displaying the effect of calendar month, the dashed red vertical line corresponds to the time Tabaski celebration.

The variables retained in the final model are indicated in Table 1 and their corresponding thin plate spline functions are graphically displayed in Figs. 4 and 5 for cattle and small ruminants respectively. Splines of variables at destination are only displayed for small ruminants in 2014 (the change in biomass at destination). In the other models the contribution of the variables at destination to the explained deviance is limited and their effect is visually very small (see supplementary Figs. S8–S11 for a full representation of the variables’ splines).

The effect of calendar months was retained in the final model for cattle in both years and for small ruminants in 2019. In the cattle model, the shape of the corresponding spline suggests an increase in the likelihood of herd movements in a 2 months period preceding the religious festival of Magal de Touba during which cattle are traditionally slaughtered. For small ruminants, the effect of calendar months was not significant in 2014 and not retained in the final model. In 2019, the calendar effect was significant and the likelihood of herd movement increased throughout the year but this increase did not seem to be related to the Tabaski celebration, during which rams are traditionally slaughtered.

The effect of biomass was retained in all final models but its pattern significantly differed between the 2014 and 2019 models. The 2014 models suggest that a lower-than-normal level of biomass production at origin in the previous month increased the likelihood of departure of cattle. Movements of small ruminants were more likely to originate from departments with intermediate levels of biomass production but where the level of biomass was decreasing, and they were more likely to be directed towards departments where biomass production was increasing from one month to the next. In 2019, any change in biomass production at the department of origin from one month to the next—positive or negative—increased the likelihood of departure, an effect observed in both cattle and small ruminants.

The effect of rainfall was retained in the final model for cattle in both years and for small ruminants in 2019. In 2014 and 2019, the likelihood of departure of cattle herds was increased by a high level of rainfall in the department of origin in the previous month, and in 2019 a reduction in the level of rainfall at the department of origin tended to decrease the likelihood of cattle herd departure. In 2019, the likelihood of departure of small ruminant herds was positively influenced by the level of rainfall in the department of origin in the previous month, although the relationship was highly nonlinear, and a reduction in the level of rainfall at the department of origin from one month to the next tended to decrease the likelihood of herd departure.

**Figure 6:**
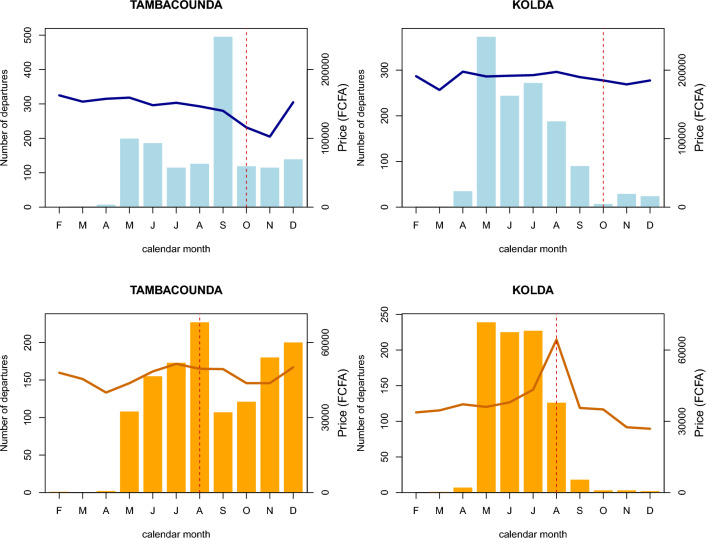
Graphical representation of the evolution of the number of registered departures of bovine and small ruminant herds (bars) and average bovine and small ruminant market price (line) in the regions of Tambacounda and Kolda in 2019. The dashed vertical red lines correspond to the months of important religious celebrations where cattle and small ruminants are traditionally consumed (Magal de Touba for cattle, Tabaski for small ruminants).

The effect of live animal market price was retained in the final model for cattle in 2019 and for small ruminants in both years. In both years, the likelihood of departure of small ruminant herds was increased by a high small ruminant market price at the department of origin in the previous month and by an increase in the same market price from one month to the next. On the contrary, in 2019, the likelihood of departure of cattle herds was decreased by high cattle market price at the department of origin in the previous month and it was increased by a decrease of cattle market price between consecutive months. This difference between the response of cattle and small ruminants’ movements to market price at origin is illustrated by the Fig. 6 showing the evolution of the aggregate price and the number of recorded herd departures in the Tambacounda and Kolda regions in 2019. The fall in cattle market price observed in Tambacounda at the end of the year 2019 is corroborated by a high number of herd departures which continues after the Magal de Touba celebration. On the other hand, high and low numbers of departures of small ruminant herds are concomitant with increases and decreases in small ruminant market prices in both Kolda and Tambacounda regions.

## Discussion

The long distance trade of live animals is expected to increase in the coming years in response to the growing demand of cities for animal proteins driven by the expanding urbanization of West African countries^11^. In addition, despite governments’ attempts at discouraging transboundary transhumant movements through border control and taxes, pastoral mobility is expected to be maintained in the coming decades to cope with the growing insecurity of the Sahel region exacerbated by climate change and political tensions^23,25,47^. In order to mitigate the sanitary effects of the expansion of livestock mobility, reliable indicators allowing a timely forecast of the short-term variations in the frequency of herd movements inside and across countries need to be identified. Rainfall and biomass production are metrics that are routinely collected over weekly intervals and at fine-scale. Live animal market prices can be recorded at monthly or weekly intervals on specific market hubs with a sufficiently high frequency of livestock transaction, enabling a continuous monitoring of market trends.

This study is a first attempt at identifying the determinants of temporal dynamics of the structure of a livestock network. Among West African countries with significant long-distance ruminant mobility, Senegal has the advantage of combining routine data collection on (1) livestock movements through the LPS system implemented by the Veterinary Services and on (2) live animal market prices by WFP. Still, these data were not collected consistently in 2014 and 2019, leading to biases in the geographical distribution of the analyzed interdepartmental links. In 2014, the links directed towards the major urban regions of Dakar and Thies were not captured in our analysis as market prices were not reported for these regions. In 2019, the movements of livestock coming from the southeast regions, where agropastoralism is predominant, were over-represented. For these reasons, the differences in results obtained between 2014 and 2019 cannot really be interpreted as an evolution in the patterns of livestock flow dynamics over this period. Another disadvantage of the used movement data is the absence of distinction between transhumance movements and commercial movement. This distinction would have enabled the building of two separate model and the identification of factors specific to each type of mobility.

From a methodological viewpoint, the use of a GAM was relevant to model non-linear effects of time-varying factors on the herd movements. This is not only true for the effect of seasonal calendar on cattle movements—which peaks near the time of religious celebrations—but also for other effects like the one of month-to-month change in biomass availability in 2019. The study makes use of different sources of data that have their limitations. Especially the herds movement data and the market price data have an incomplete geographical coverage or a high number of missing observations. The use of a hurdle Poisson model conveniently addressed the patchy nature of the movement data, the high frequency of absence of movement notification being a likely consequence of under-reporting to authorities rather than true absence of movements^5^. In order to assess the robustness of the model results, it would be necessary to reproduce the analysis on either (1) data from the same sources collected in later years—the collection of herd movement records and market prices is still an ongoing process in Senegal—or (2) data from a different source, for example a field survey conducted in one or two representative subpopulations of ruminant farmers or traders of Senegal, which would include the longitudinal collection of herd movement data and market price data.

The largest fraction of the variance in the frequency of herd movements was explained by geographical determinants that do not vary in time—i.e. intrinsic properties of the interdepartmental links. This is consistent with the existing literature on pastoralism: commercial movements are typically directed from livestock production regions—north and southeast of Senegal—to consumption hubs, located on the coastal regions, especially the major urban center of Dakar^22^. Meanwhile, a large fraction of the transhumance movements connects northern and southern regions and transhumant farmers tend to use well known itineraries. Nevertheless, the network structure and frequencies of herd movements displayed substantial variation along the years 2014 and 2019 and the fitted models explained between 17 and 71% of this variation depending on year and species. Time-varying variables at the departments of origin had a substantially higher contribution to the model fit compared to time-varying variables at destination and calendar. Decisions to sell animals or to move herds are likely based on what farmers observe in their direct environment, including the availability of pasture and market prices, while the direction of movements tend not to vary substantially over time, as farmers and traders prefer to use well-known paths and resting points, and relocate herds or sell animals in places they are familiar with^23^.

The effect of calendar, related to the occurrence of religious celebrations, was significant for cattle but very weak or not significant for small ruminants, which is surprising given the high reported impact of the Tabaski festival on the demand and consumption of small ruminants^22^. However, the Tabaski celebration coincided with high live sheep market prices, which had a strong positive effect on small ruminant herd departures in our models. While small ruminant farmers plan their production to supply a high number of rams near the time of Tabaski, they seem to keep their animals until the market price at their location is sufficiently high, a speculative behavior which may explain the high price peak observed at the exact time of Tabaski. This effect was not found for cattle, for whom the likelihood of movement was increased in the two months preceding the Magal celebration, independently of other variables, which may explain why the cattle price increase attributed to Magal is relatively less pronounced. Instead price falls seem to have caused a high number of cattle departures in 2019. This difference in speculative behavior should be elucidated with field surveys targeting farmers and traders. One hypothetical explanation is the lower cost of keeping sheep, in terms of feed consumption, as compared with cattle, which makes it easy for farmers to maintain a high number of sheep in their herd until prices reach a sufficiently high level, and much less so for cattle.

In all years and species, except small ruminants in 2014, the model outputs suggest a positive effect of the level of rainfall at origin on the likelihood of herd movement. According to Adriansen^24^, one of the reasons of pastoral mobility is a southward transhumance aimed at moving herds to places where the rain is expected to start first, which means farmers may plan their journey back to the northern areas when the rainfalls have already started or have reached a high level. Due to the limited data availability, it was not possible to determine whether the effect of rainfall was specific to movements directed northward. The effect of biomass production differed between study years. In 2014, cattle and small ruminant departures were seemingly driven by a low level or decreasing level of biomass production in the place of origin and, in the case of small ruminants, an increase in biomass production at destination. This is consistent with a mobility aimed at finding better grazing areas or at selling animals to reduce feed requirements in case of scarcity of pasture. In 2019, increases as well as decreases in biomass production at the place of origin increased the likelihood of departure, indicating a more complex causal relationship. According to Turner and Shlecht^23^ the extension of cultivated areas tends to increase livestock mobility because cultivation produces local forage scarcity, particularly in the rainy season, and risks of conflicts with crop producers. We can hypothesize that an increase in biomass production could be indicative of the growth of cultivated crops, which increases the risk of such conflicts and motivates the departure of livestock herds. This effect was only revealed with the 2019 dataset because of the over-representation of movements originating in the southeastern “agropastoral” region, combining livestock farming and cultivation.

Environmental variables displayed a consistent pattern of variation across departments and years of study. The variation in rainfall and biomass production is primarily determined by the alternation of the dry and rainy seasons, although with some differences in the exact month of increase in rainfall and biomass production across departments. Live animal market prices exhibited a much more heterogeneous pattern of variation and, in the case of cattle, there was no obvious spatial structure in the distribution of these different price variation patterns, as markets with similar patterns were not located significantly closer. According to Turner et al. live animal market prices are influenced by the state of supply and demand for livestock products at the time of the transaction, but biological characteristics of the animals and season also have an influence, and emergency sales of animals, in situation of scarcity of food or disease outbreaks, are performed at a below-average price^20^. This probably explains the price fall on June 2014, which corresponded to the end of the dry season, when the shortage of feed and the body condition of animals are at their worst—the so-called “hunger gap” period. Religious celebrations, during which the demand for livestock meat is high, coincided with an increase in market prices, especially Tabaski.

The model results suggest that monitoring variation in biomass production is of potential interest for forecasting departures of both cattle and small ruminant herds from their grazing areas, while cattle commercial movements significantly increase in the two months period preceding the Magal de Touba celebration. This information is useful for guiding future surveillance and control efforts for limiting the risk of disease spread caused by livestock mobility. While religious celebrations and vegetation follow a regular and predictable pattern, this is not the case of market price. The substantial effect of market price on small ruminant herd departures, evidenced by the model results, added to the high unpredictability of the market price evolution, suggest an added value of complementing current animal health surveillance systems with a regular recording of live animal prices. This recording should be performed at defined short intervals, for example once per week, in livestock markets located in the main ruminant production areas of Senegal. This monitoring could enable forecasting the peaks of long-distance flows of small ruminants motivated by commercial purposes and associated risk of disease propagation from production areas where the price increases towards other locations. This is especially true given the role played by the long distance movements of goats and sheep in the dissemination of at least two major TIDs, Rift Valley Fever^19^ and Peste des Petits Ruminants^1^.

## Conclusion

Livestock herd movements initiated by farmers and traders are mainly motivated by a maximization of the revenues from animal sales and a limitation of feed costs. In consequence, availability of pasture, market prices and religious festivals, during which the demand for livestock is high, are responsible for a substantial fraction of the dynamics of the livestock network structure. The study demonstrates the potential benefits of routine collection of livestock movements and market prices information for future efforts aimed at forecasting variations in the spatial distribution of livestock movements and a better anticipation and management of the long-distance transmission of contagious diseases. The analysis performed in the study should be reproduced in other countries and on other time periods to assess the robustness of the findings, the reliability of the environmental and economic indicators and their potential use for future attempts at forecasting livestock movements and infectious disease outbreaks.

### Supplementary Information

Supplementary Figures.

## Data Availability

Rainfall and biomass production data are public and accessible at the GPCC^35^ and Copernicus^36^ website respectively. Market prices and livestock mobility data are the property of the World Food Program and the national Veterinary Services of Senegal respectively and are not publicly available. The datasets can be obtained from the authors upon request and with the permission of the concerned institution.
