# Supplementary material for: Environmental and economic determinants of temporal dynamics of the ruminant movement network of Senegal
Source: Sci Rep. 2023 Sep 2;13:14482. doi: 10.1038/s41598-023-40715-3 (PMC10475130; doi:10.1038/s41598-023-40715-3)
Supplement: Supplementary file 1 — Supplementary Figures. [file 41598_2023_40715_MOESM1_ESM.pdf]

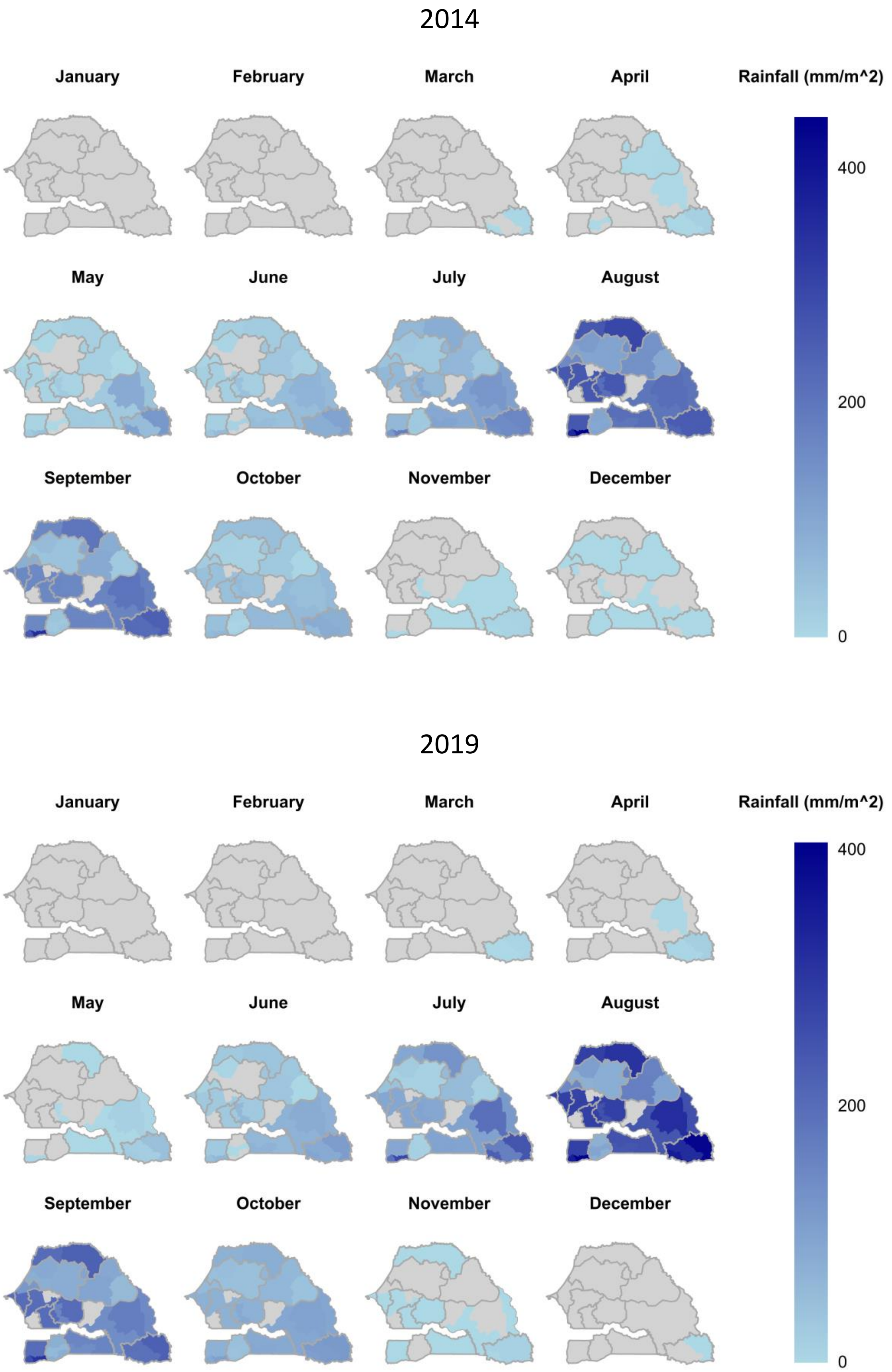

Figure S1. Spatial representation of the monthly rainfall measures in the administrative departments of Senegal in years 2014 (top) and 2019 (bottom). The color intensity of each department is proportional to the measure of rainfall in the corresponding month and gray color means that no rainfall was recorded. Dark lines correspond to region borders.

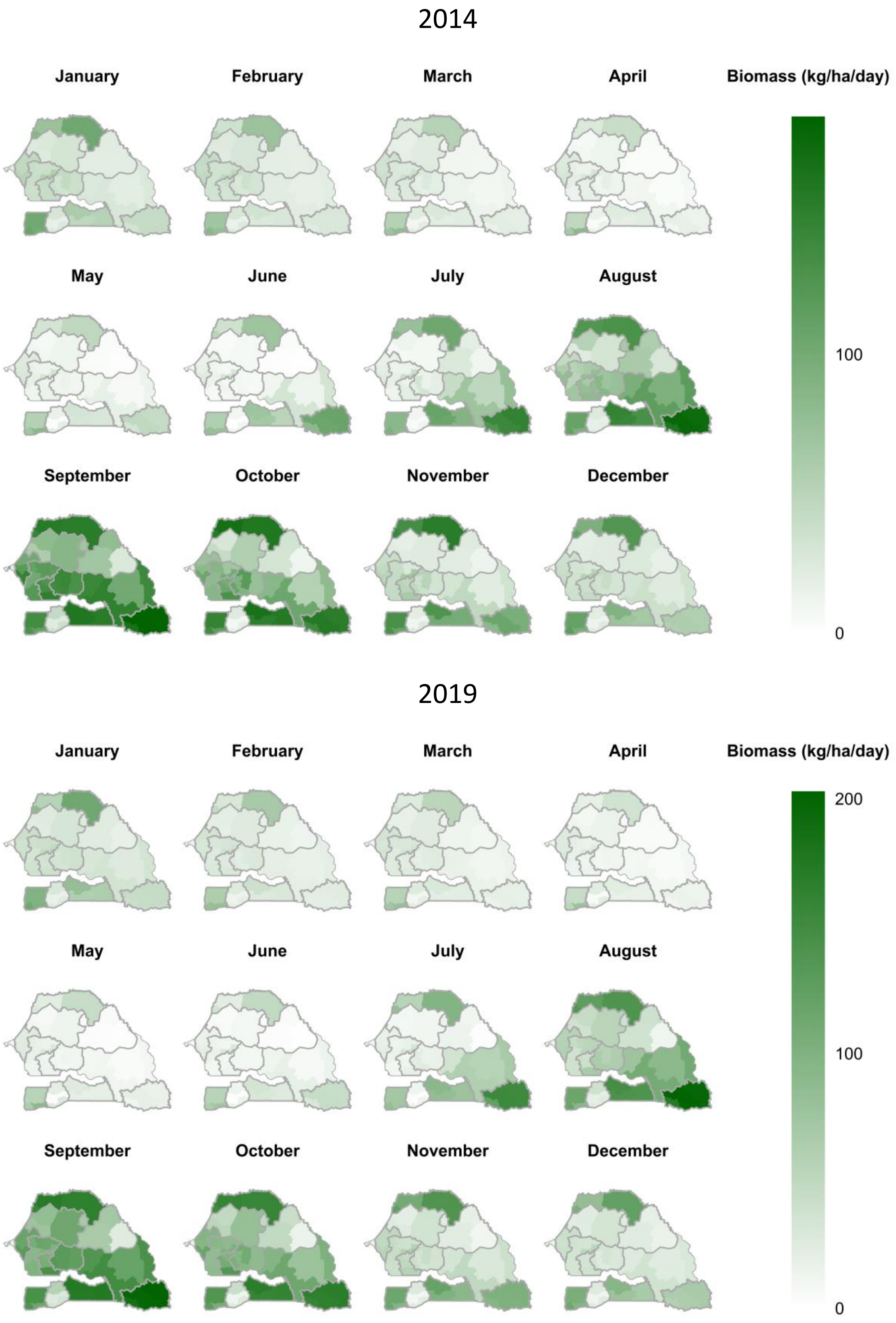

Figure S2. Spatial representation of the monthly biomass production measures in the administrative departments of Senegal in years 2014 (top) and 2019 (bottom). The color intensity of each department is proportional to the measure of biomass production in the corresponding month. Dark lines correspond to region borders.

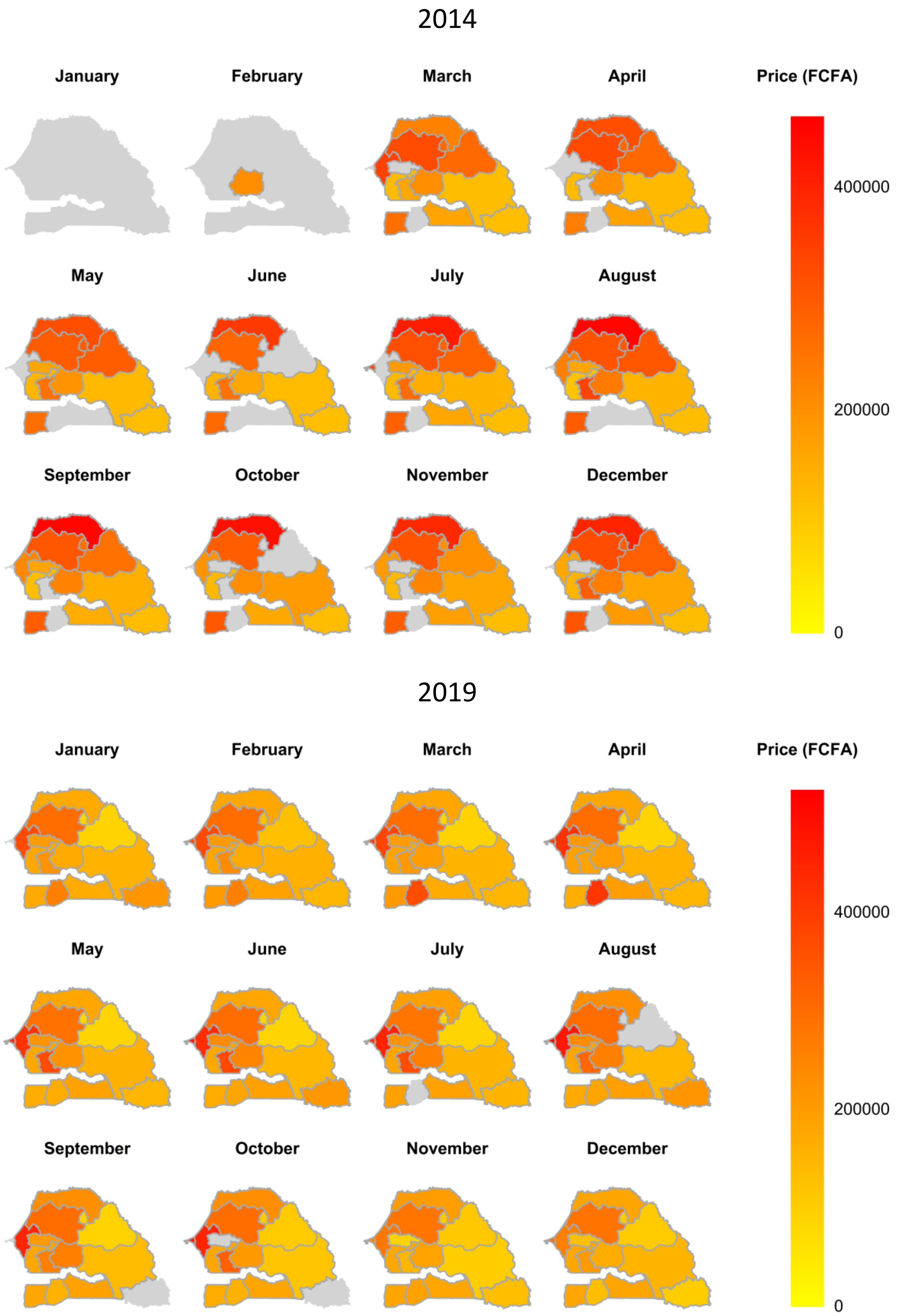

Figure S3. Spatial representation of the monthly average cattle market prices in the administrative regions of Senegal in years 2014 (top) and 2019 (bottom). The color intensity of each region is proportional to the measure of biomass production in the corresponding month. Dark lines correspond to region borders. Gray color indicates missing observations.

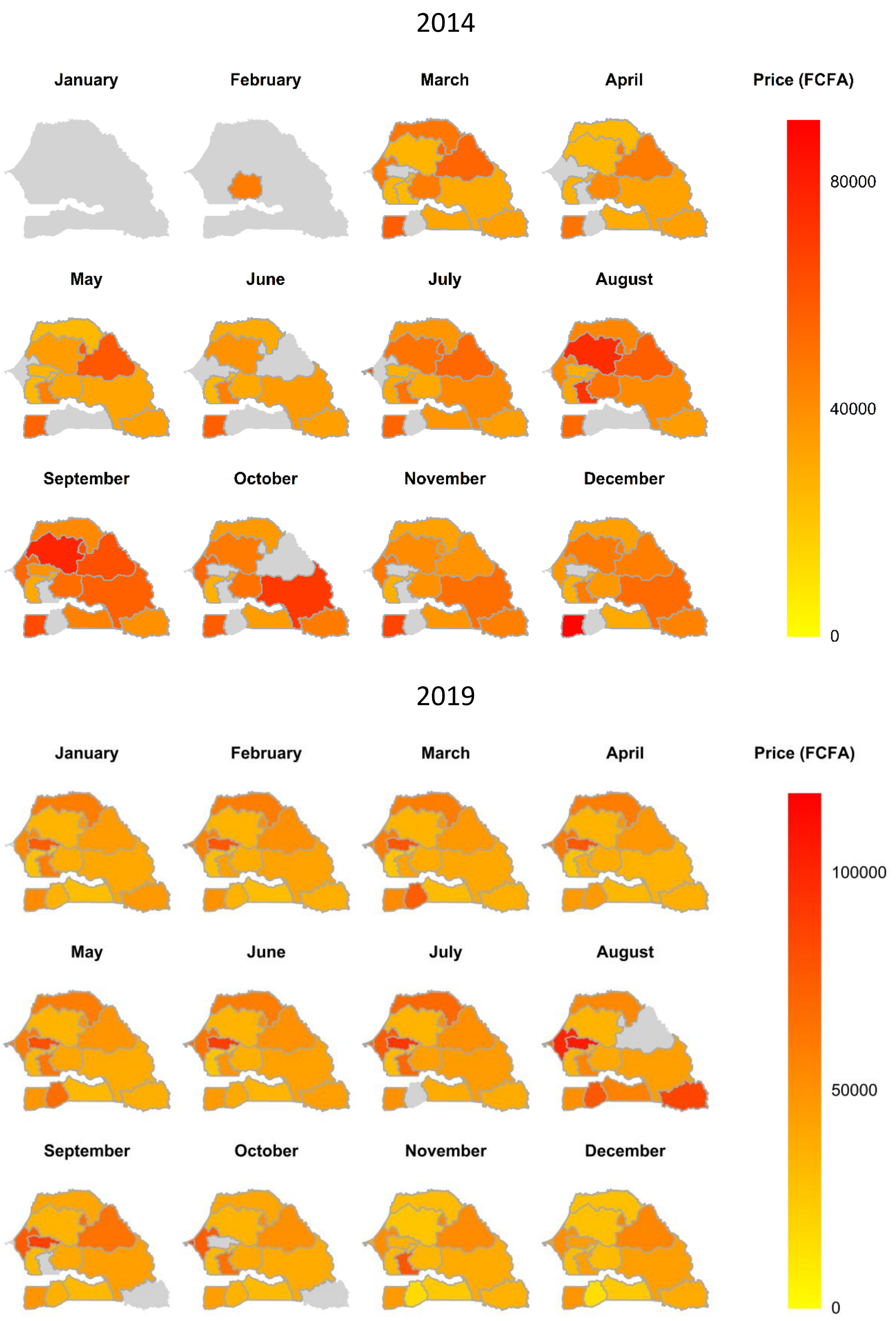

Figure S4. Spatial representation of the monthly average small ruminant market prices in the administrative regions of Senegal in years 2014 (top) and 2019 (bottom). The color intensity of each region is proportional to the measure of biomass production in the corresponding month. Dark lines correspond to region borders. Gray color indicates missing observations.

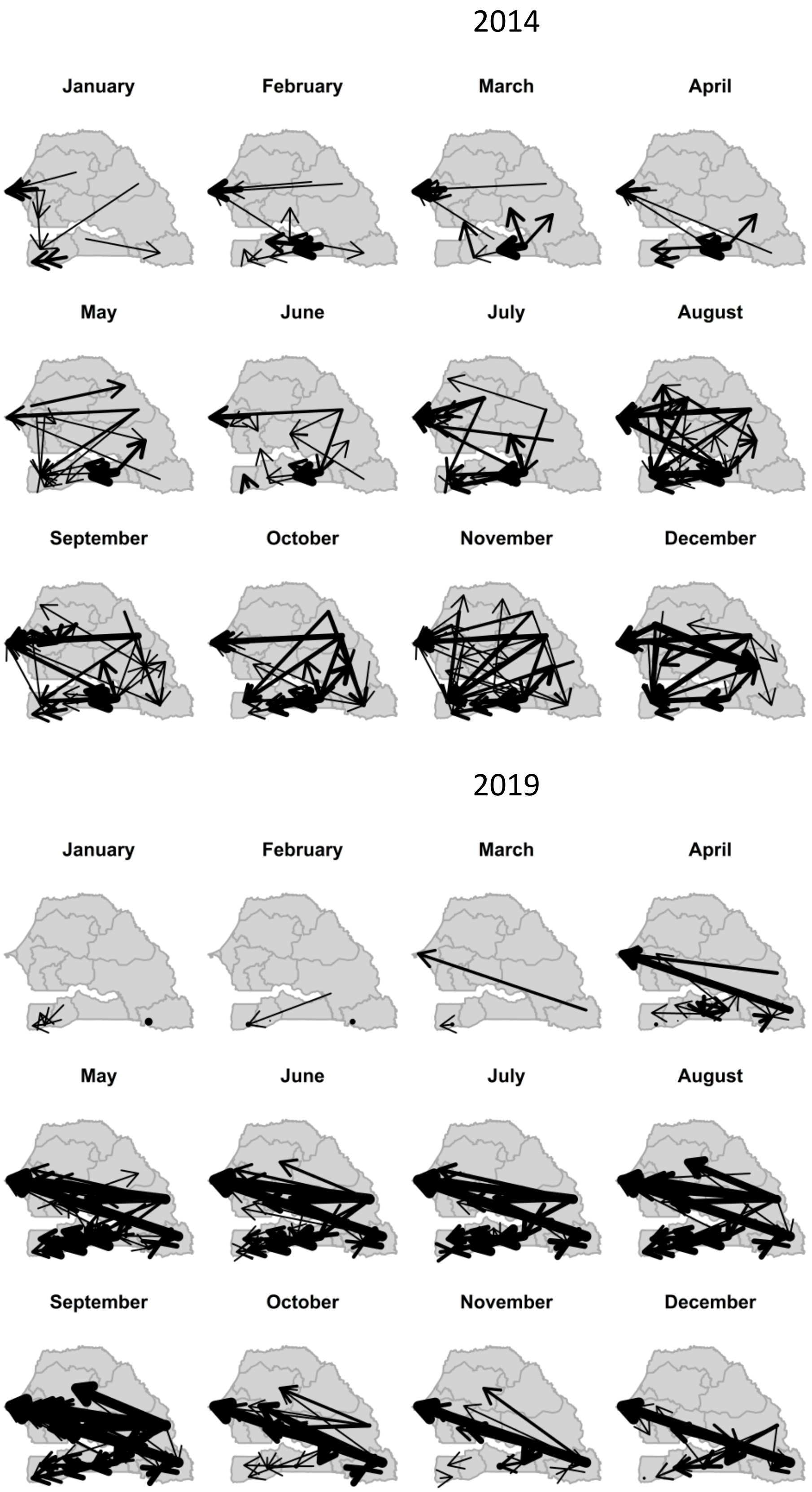

Figure S5. Spatial representation of the monthly evolution of the number of recorded movements of cattle herds between administrative departments of Senegal in years 2014 (top) and 2019 (bottom). Directed arrows indicate a non-null number of movements recorded between departments of origin and destination. Arrow width is proportional to the logarithm of the number of recorded herd movements. Dark lines correspond to region borders.

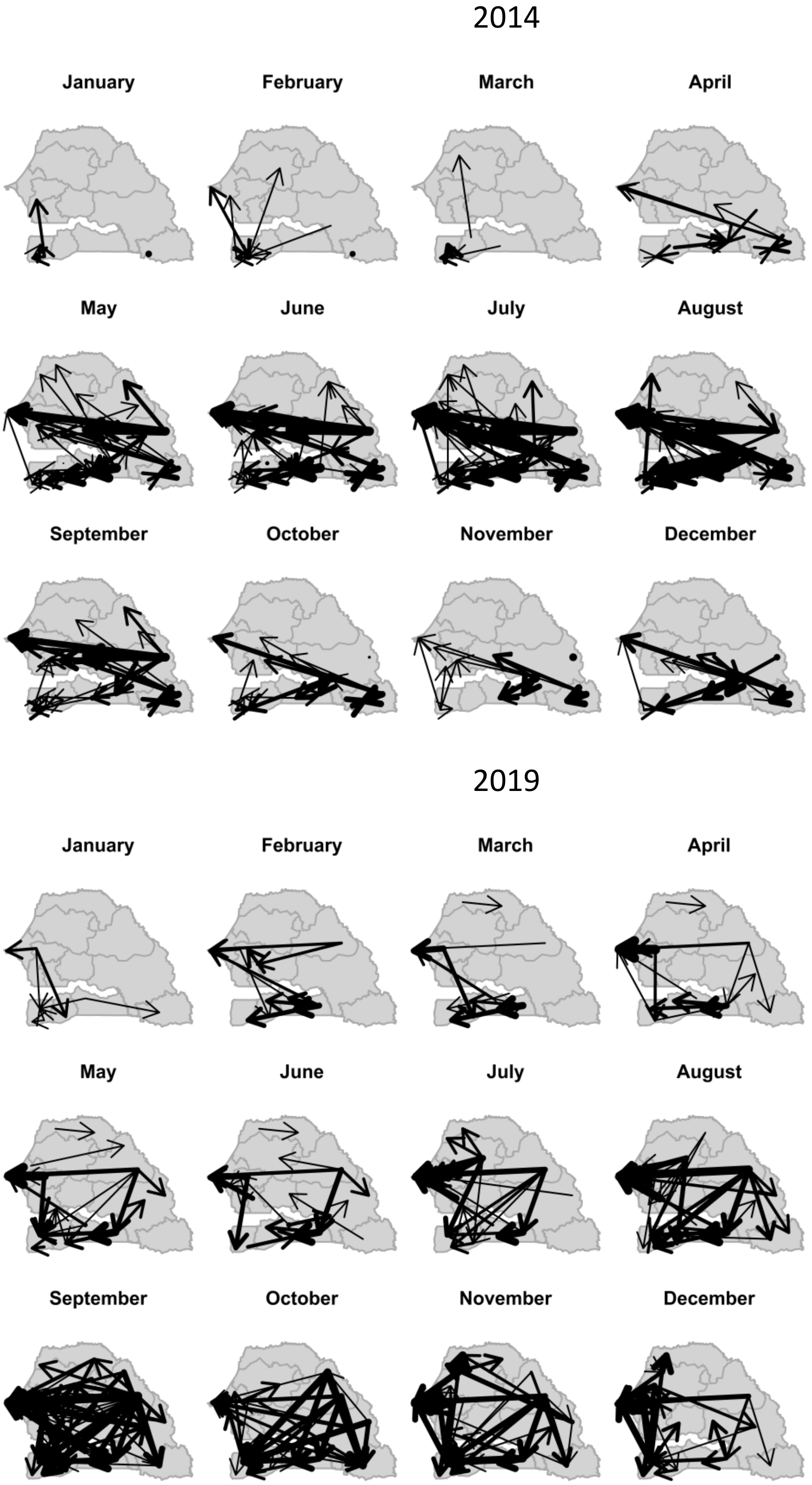

Figure S6. Spatial representation of the monthly evolution of the number of recorded movements of cattle herds between administrative departments of Senegal in years 2014 (top) and 2019 (bottom). Directed arrows indicate a non-null number of movements recorded between departments of origin and destination. Arrow width is proportional to the logarithm of the number of recorded herd movements. Dark lines correspond to region borders.

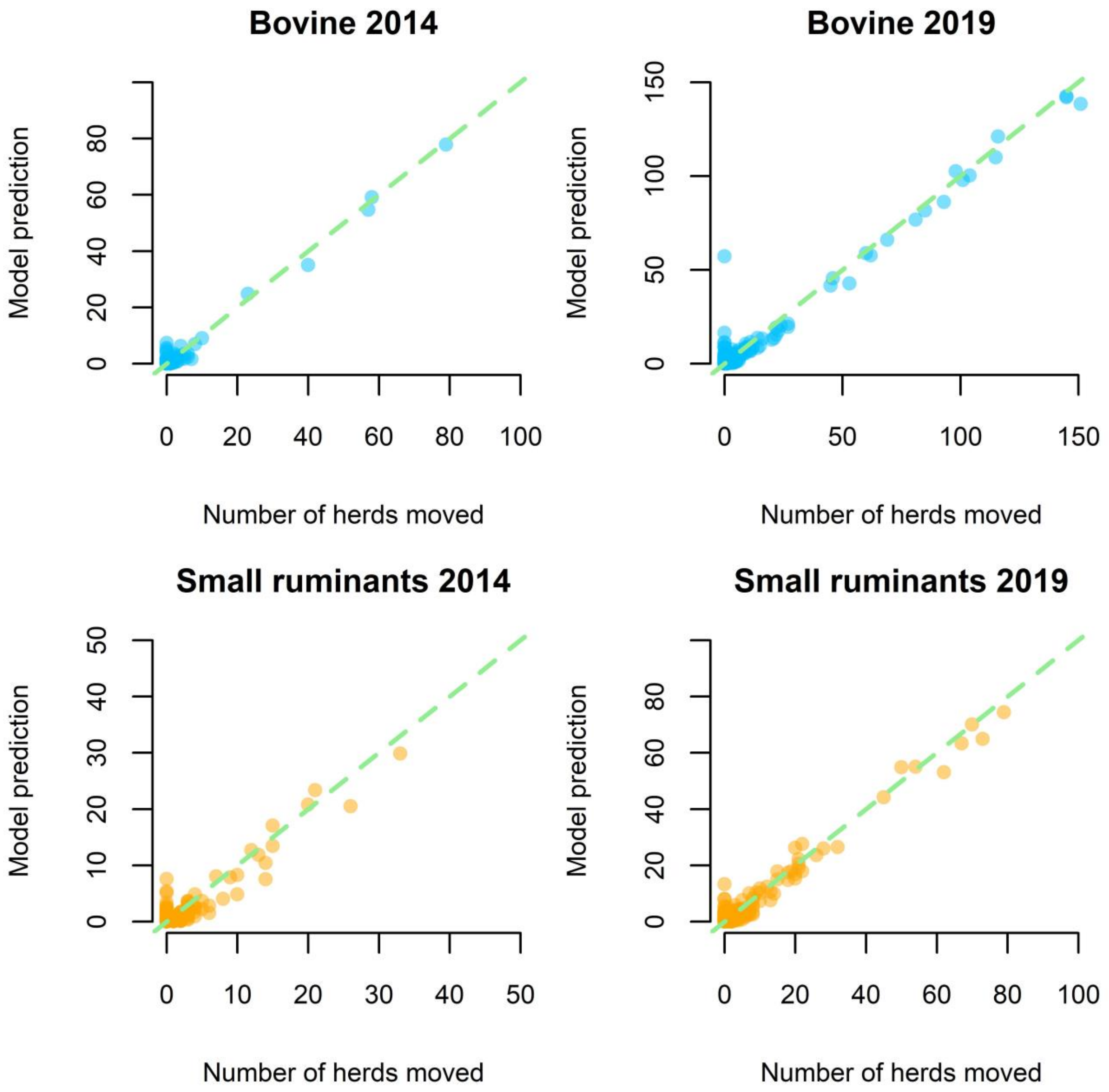

Figure S7. Dot plot representation of the relationship between number of recorded herd movements between pairs of departments at specific months and corresponding final model predictions. The dashed green line is the identity line.

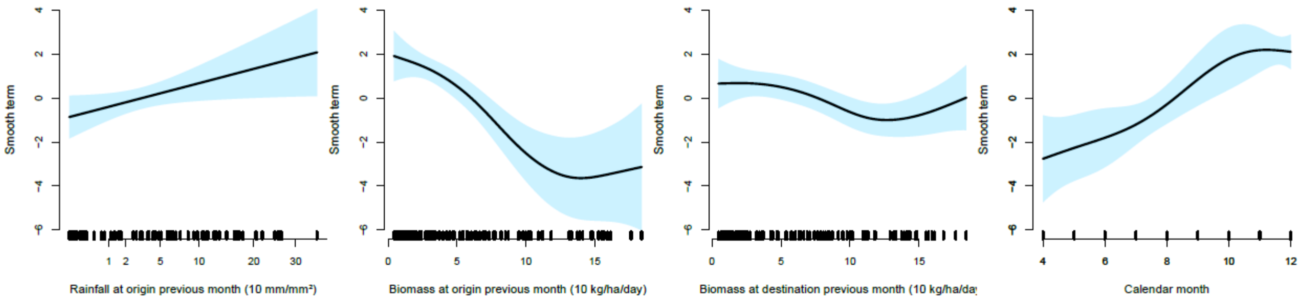

Figure S8. Graphical representation of the fitted thin plate spline functions of the effect of tested variables on the likelihood of bovine herd movement along a defined inter-department link on a defined month in 2014. Black lines are spline functions linking the tested variable to the linear predictor, and blue bands correspond to the 95% confidence interval.

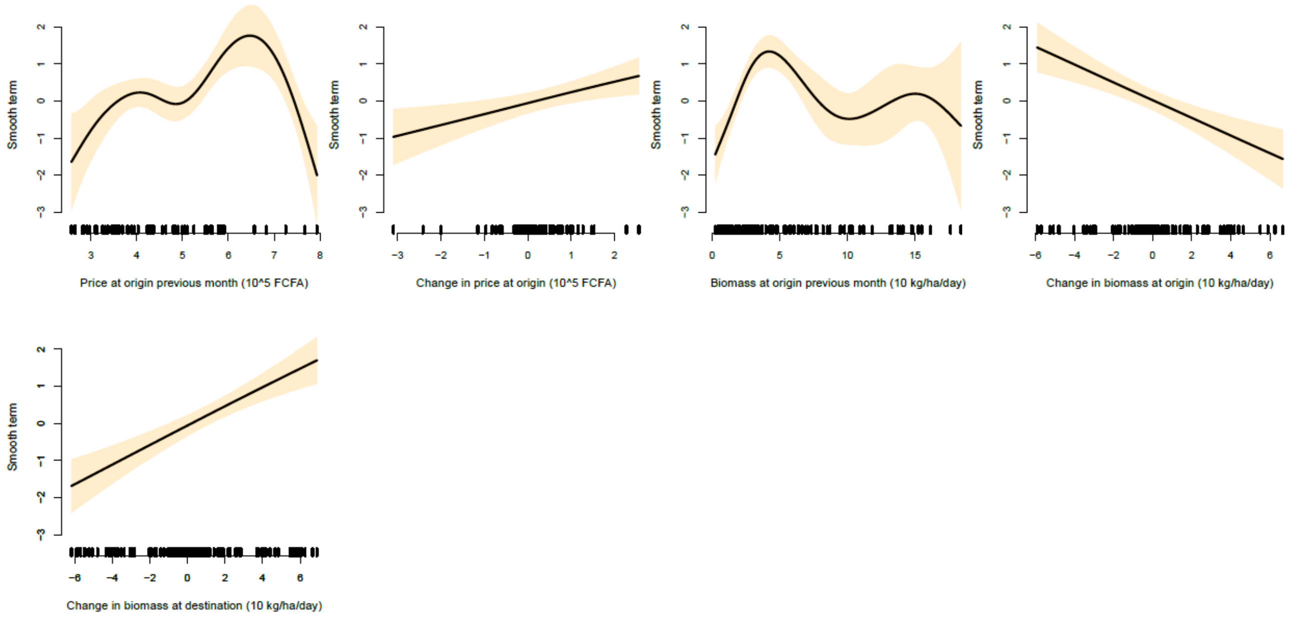

Figure S9. Graphical representation of the fitted thin plate spline functions of the effect of tested variables on the likelihood of small ruminant herd movement along a defined inter-department link on a defined month in 2014. Black lines are spline functions linking the tested variable to the linear predictor, and blue bands correspond to the 95% confidence interval.

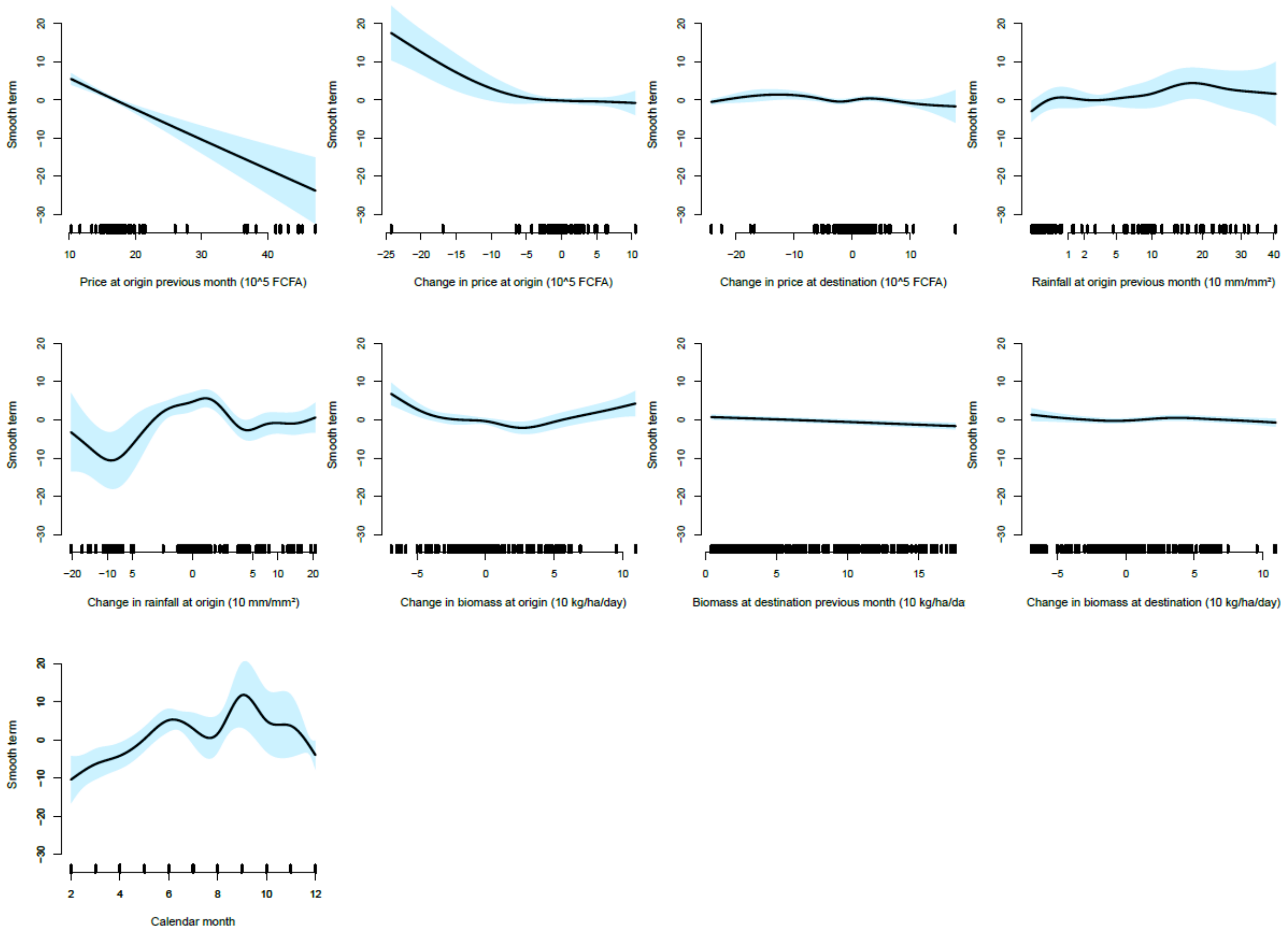

Figure S10. Graphical representation of the fitted thin plate spline functions of the effect of tested variables on the likelihood of bovine herd movement along a defined inter-department link on a defined month in 2019. Black lines are spline functions linking the tested variable to the linear predictor, and blue bands correspond to the 95% confidence interval.

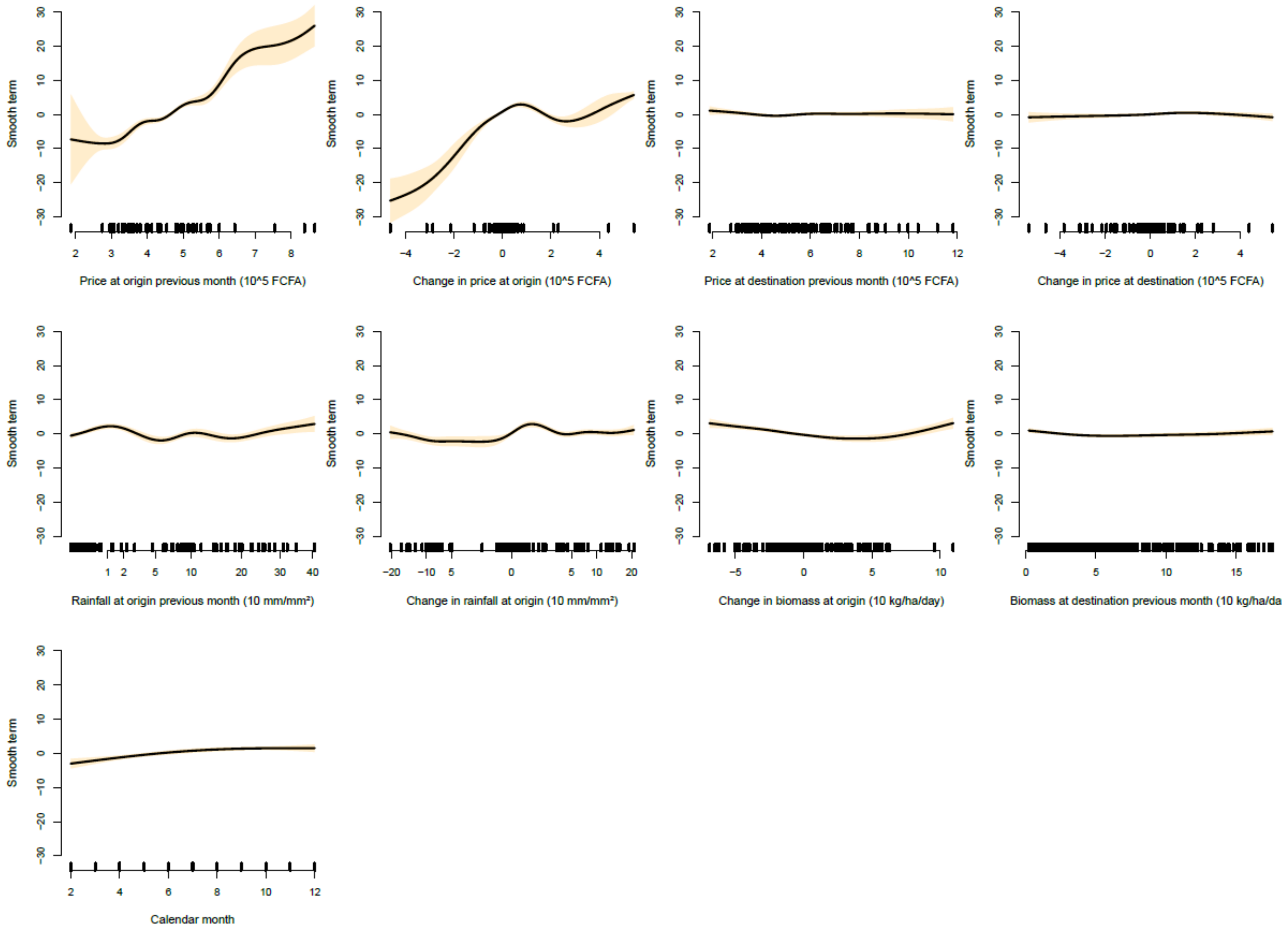

Figure S11. Graphical representation of the fitted thin plate spline functions of the effect of tested variables on the likelihood of small ruminant herd movement along a defined inter-department link on a defined month in 2019. Black lines are spline functions linking the tested variable to the linear predictor, and blue bands correspond to the 95% confidence interval.
